# Serum Levels of Stress Hormones and Oxidative Stress Biomarkers Differ according to Sasang Constitutional Type

**DOI:** 10.1155/2015/737631

**Published:** 2015-10-11

**Authors:** Hyeong Geug Kim, Yoon Jung Kim, Yo Chan Ahn, Chang Gue Son

**Affiliations:** ^1^Liver and Immunology Research Center, Daejeon Oriental Hospital of Daejeon University, 176-9 Daeheung-ro, Jung-gu, Daejeon 302-724, Republic of Korea; ^2^Department of Health Service Management, Daejeon University, 62 Daehak-ro Yongun-dong, Song-gu, Daejeon 300-716, Republic of Korea

## Abstract

*Objectives.* This study investigated whether Sasang constitutional type is associated with differences in the serum levels of stress hormones and oxidative stress.* Methods.* A total of 236 participants (77 males and 159 females) were enrolled. The serum levels of cortisol, adrenaline, reactive oxygen species (ROS), and malondialdehyde (MDA) were analyzed.* Results.* The distribution of Sasang constitutional types was as follows: Taeumin, 35.6%; Soumin, 33.0%; and Soyangin, 31.4%. The serum cortisol levels of Taeumin were significantly lower than Soumin (*p* < 0.1 in both sexes) and Soyangin (*p* < 0.05 in males and *p* < 0.1 in females). The adrenaline levels were also significantly lower in Taeumin than in Soumin (*p* < 0.05 in males and *p* < 0.1 in females) and Soyangin (*p* < 0.1 in males). Serum ROS levels were significantly higher in Soyangin than in Taeumin and Soumin (*p* < 0.05 in males), whereas MDA levels were significantly lower in Taeumin compared with Soumin and Soyangin (*p* < 0.05 in males and *p* < 0.1 in females).* Conclusion.* Taeumin type may tolerate psychological or oxidative stress better than other types, which suggests a biological mechanism to explain the different pathophysiological features of Sasang constitutional types.

## 1. Introduction

Sasang constitutional medicine (SCM) is a major branch of traditional Korean medicine that emphasizes the role of inherited psychological and physical traits in the development and treatment of diseases [1]. SCM was established and popularized by Lee (1837–1900), a Korean doctor, through his book* Donguisusebowon* [2], and it has since been widely adopted as a diagnostic and therapeutic tool in traditional Korean medicine [3].

SCM classifies people into four types according to their constitution: Taeyangin, Soyangin, Taeumin, and Soumin. In this context, “constitution” includes the body's structural and functional features, including psychological characteristics [4]. Current genetic science has shown that the genome critically affects the complex processes involved in health and disease [5], and the SCM classification is presumably related to macrolevel genomic differences among individuals [6]. Moreover, recent studies have found that certain Sasang types are significantly more susceptible to certain diseases; specifically, those with the Taeumin type are especially susceptible to metabolic syndrome, and those with the Soumin type are especially susceptible to irritable bowel syndrome (IBS) [7, 8].

Psychological or emotional stress, which is inevitable in modern life, is associated with both mental and physical health [9]. Moreover, psychological characteristics have been associated with Sasang constitutional types [10]. In addition, evidence has shown a strong connection between psychological stress and oxidative stress, prominent features of pathophysiological processes in a wide range of disorders or aging [11, 12]. Therefore, we hypothesized that Sasang constitutional type would affect the serum levels of stress hormones, especially cortisol and adrenaline, and oxidative stress biomarkers including reactive oxygen species (ROS) and its lipid oxidation byproduct.

To contribute to elucidating the biological explanation for SCM, we initially investigated whether the serum level of stress hormones and the degree of oxidative stress differ according to Sasang constitution.

## 2. Methods

### 2.1. Subjects

Self-reported healthy adults were recruited in South Korea, and those who had an established illness or were taking medication were excluded from the study. This study also excluded night workers, alcoholics, and subjects with self-reported severe psychological stress. Blood samples were collected at least 4 hours after participants' last meal before noon. Informed consent was obtained from each subject and the Ethics Committee of Daejeon University Hospital approved the study protocol (authorization number: DJOMC-119).

### 2.2. Classification of Sasang Constitution

The grouping of participants according to type of Sasang constitution was based on consideration of an integrative combination of facial, body shape, vocal, and questionnaire response features using Sasang constitutional analytic tool (SCAT). This automated Sasang constitution classification system developed by the Korea Institute of Oriental Medicine [13]. Every participant was classified as Taeumin, Soumin, Taeyangin, or Soyangin.

### 2.3. Determination of Serum Cortisol and Adrenaline Levels

Serum cortisol and adrenaline levels were determined using a cortisol ELISA kit (LDN GmbH & Co., KG, Nordhorn, Germany) and an adrenalin ELISA kit (LDN GmbH & Co, KG, Nordhorn, Germany), respectively, according to the manufacturer's protocol. Absorbance was measured using a spectrophotometer (Molecular Devices, Sunnyvale, CA).

### 2.4. Determination of Serum ROS Levels

The total quantity of ROS in the serum was determined according to Hayashi' method [14]. Briefly, N,N-diethyl-para-phenylenediamine (DEPPD) and ferrous sulfate solutions were prepared in advance. Five *μ*L of standard solution or serum was added to 140 *μ*L of 0.1 M sodium acetate buffer (pH 4.8) in 96-well plates and incubated at 37°C for 5 min. A total of 100 *μ*L of DEPPD and ferrous mixture solution was added to each well, and the amount of ROS was determined at the saturation point at 505 nm using a spectrophotometer. Hydrogen peroxide was used to generate the calibration curve, and the standard and the results were expressed as equivalent to levels of hydrogen peroxide (*μ*mol H_2_O_2_ equiv/L).

### 2.5. Determination of Serum Malondialdehyde (MDA) Levels

Serum lipid peroxide levels were determined using thiobarbituric acid (TBA) reactive substances (TBARS) as previously described [15]. Briefly, 250 *μ*L of serum or standard solution was added to 2.5 mL of 20% trichloroacetic acid (TCA). This was then mixed with 1 mL of 0.67% thiobarbituric acid (TBA) and heated at 10°C for 30 min, followed by cooling on ice. After centrifugation at 3000 ×g for 20 min, the absorbance of the upper organic layer was measured at 535 nm with a spectrophotometer and compared with a 1,1,3,3-tetraethoxypropane (TEP) standard curve.

### 2.6. Statistical Analysis

Statistical analysis was performed using SAS statistical software (SAS Rel. 8.02; SAS Institute, Inc., Cary, NC, USA). Comparisons among Sasang constitution groups were performed by one-way ANOVA followed by paired Student's *t*-tests. *p* values < 0.1 were considered statistically significant. All data are expressed as means ± standard deviations (SDs).

## 3. Results

### 3.1. Characteristics of Participants

A total of 236 adults, including 77 males (median age: 23 yr, range: 18–63) and 159 females (median age: 21 yr, range 18–77), participated in this study. The proportions of Taeumin, Soumin, and Soyangin among total participants were 35.6%, 33.0%, and 31.3% respectively. The sample included no participants with a Taeyangin constitution. Among males, 23.4%, 45.5%, and 31.2% were of Taeumin, Soumin, and Soyangin constitution, respectively; among females, 41.5%, 27.0%, and 31.5% were of Taeumin, Soumin, and Soyangin constitution, respectively (Table 1).

The average heights of the three Sasang types were similar; however, the body weights of differed notably among categories. The average body mass index (BMI) differed significantly according to Sasang constitution among male, female, and total participants (*p* < 0.01). The BMI of those with a Taeumin constitution was significantly higher than that of participants with a Soumin (*p* < 0.01 in male, female, and total subjects) or a Soyangin (*p* < 0.01 in male, female, and total subjects) constitution, and the BMI of those with a Soumin constitution was significantly lower than that of participants with a Soyangin constitution (*p* < 0.05 in male and total subjects, Table 1).

### 3.2. Serum Levels of Cortisol

**Figure 1:**
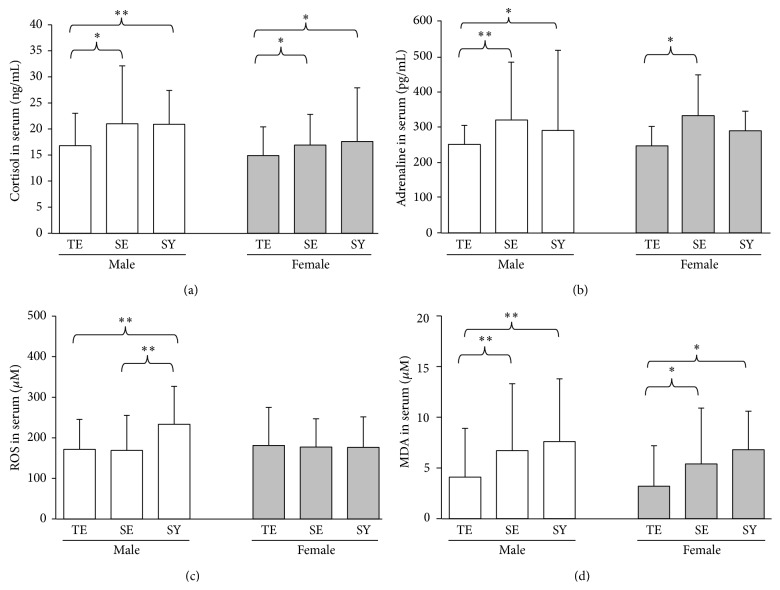
Serum levels of stress-related hormones and oxidative stress biomarkers. Blood was collected from healthy adults. The serum levels of cortisol (a) and adrenalin (b), reactive oxidative species (ROS (c)), and malondialdehyde (MDA (d)) were measured. Results are expressed as means ± SDs. Statistical significance was set at ^∗^ *p* < 0.1 and ^∗∗^ *p* < 0.05.

The serum cortisol level of males was significantly higher than that of females (20.0 ± 8.9 versus 16.3 ± 7.5 ng/mL, *p* = 0.002). Males with a Taeumin constitution (16.8 ± 6.2 ng/mL) had a significantly lower serum cortisol level than those with a Soumin (21.0 ± 11.1 ng/mL, *p* = 0.091) or a Soyangin (20.9 ± 6.5 ng/mL, *p* = 0.049) constitution. This pattern was repeated in female: Taeumin (14.9 ± 5.5 ng/mL) comparing to Soumin (16.9 ± 5.9 ng/mL, *p* = 0.081) and Soyangin (17.6 ± 10.3 ng/mL, *p* = 0.096), respectively (Figure 1(a)). No significant difference in serum cortisol was observed between Soumin and Soyangin constitutional groups (*p* = 0.685).

### 3.3. Serum Adrenaline Levels

The serum adrenaline level of males was slightly higher than that of females (296.1 ± 126.4 versus 284.4 ± 85.4 pg/mL, *p* = 0.604). Males with a Taeumin constitution (250.9 ± 50.4 pg/mL) had a significantly lower level of serum adrenaline than males with a Soumin (321.3 ± 165.3 ng/mL, *p* = 0.027) or Soyangin (291.0 ± 230.9 pg/mL, *p* = 0.069) constitution. A statistically significant difference was observed between Taeumin (246.9 ± 55.4 pg/mL) and Soumin (333.2 ± 118.0 ng/mL, *p* = 0.084), but Soyangin (290.3 ± 54.4 ng/mL, *p* = 0.198), for serum adrenaline in females (Figure 1(b)).

### 3.4. Serum ROS Levels

Males and females had almost identical serum ROS levels overall (178.6 ± 81.4 versus 189.3 ± 89.8 *μ*M, *p* = 0.382). Soyangin males (233.3 ± 93.5 *μ*M) had a significantly higher level than Taeumin (171.5 ± 73.9 *μ*M, *p* < 0.05) and Soumin (168.9 ± 86.5 *μ*M, *p* < 0.05) males, but no significant differences were observed in females (Taeumin 181.0 ± 93.8 versus Soumin 177.2 ± 69.8 versus Soyangin 176.5 ± 75.0 *μ*M, resp., Figure 1(c)).

### 3.5. Serum MDA Levels

The overall serum MDA levels of males and females were very similar (6.4 ± 5.6 versus 6.0 ± 6.8 *μ*M, *p* = 0.415). Taeumin males (4.1 ± 4.8 *μ*M) had a significantly lower value compared with Soumin (6.7 ± 6.6 *μ*M, *p* = 0.039) and Soyangin (7.6 ± 6.2 *μ*M, *p* = 0.040) males, and this pattern was repeated in females (Taeumin: 3.2 ± 4.0 *μ*M; Soumin: 5.4 ± 5.5 *μ*M, *p* = 0.081; Soyangin: 6.8 ± 3.8 *μ*M, *p* = 0.062, Figure 1(d)). No significant difference was observed between Soumin and Soyangin constitutions in males (*p* = 0.594) or females (*p* = 0.732).

## 4. Discussion

Inherited genomic variance is a critical contributor to individual differences in the development of various diseases [16]. SCM stresses the importance of inborn physical and psychological characteristics, which might be linked to macrogenomic features [17, 18]. Several studies have found that the high incidence of certain disorders and different drug responses are associated with Sasang constitution-dependent genomic features, likely the significantly different distributions of Pro12Ala polymorphism or haplotypes of multidrug resistance 1 (MDR1) gene [19, 20].

We examined whether the serum levels of two representative stress hormones and oxidative stress markers differ according to Sasang constitution. Psychological stress and oxidative stress have been established as general contributors to various disorders, including cancer and immunological and age-related diseases [21, 22]. The total proportions of Taeumin, Soumin, and Soyangin constitutional types in this sample were 35.6%, 33.0%, and 31.3%, respectively (Table 1). No participant was classified as Taeyangin, probably because this type is extremely rare in the Korean population [23].

Taeumin males and females showed relatively lower concentrations of serum cortisol and adrenaline compared with Soumin and Soyangin males and females (Figures 1(a) and 1(b)). Cortisol and adrenaline are considered typical stress hormones that are released via activation of the hypothalamic–pituitary–adrenal (HPA) axis under stress [9]. These stress hormones have a harmful effect on multiple target organs and systems, with outcomes including chronic pain, immunosuppression, and psychological disorders [24, 25]. Our results suggest a partial explanation of clinical data showing that Soumins are vulnerable to stress and irritable bowel syndrome (IBS) [8]. Individuals with IBS were known to have the sustained HPA axis activity [26], and then Soumins showed the highest levels of both cortisol and adrenaline in our study. Additionally, activation of the HPA axis is associated with acceleration in oxidative stress via unbalanced redox, including excessive production of mitochondrial ROS [27, 28]. In our study, the serum levels of ROS and its lipid peroxidation product MDA were low-high in Taeumins but high in Soumins and Soyangins (Figures 1(c) and 1(d)). Oxidative stress is implicated in diverse pathophysiological conditions, including inflammation, neurodegenerative diseases, and cancer [29]. Our previous study found that the incidence of cancer was significantly lower in Taeumins than in Soumins or Soyangins [30]. The serum cortisol levels however would be affected by time point of blood sample because circadian rhythm in the release of cortisol and adrenaline is well known [31, 32].

The analysis of physical features showed that BMIs differed significantly according to Sasang constitutional type; those with the Taeumin type had the highest BMIs, followed by those with the Soyangin type and those with the Soumin type (Table 1), which is consistent with results from other researches [33]. BMI is well known as a risk factor for type 2 diabetes; however, one study found that Taeumin constitution is an independent risk factor for this disease regardless of BMI score [34]. In our study, BMI values did not reflect a pathogenic level; the maximum value was 27.3 ± 2.2 (in Taeumins), and the minimal value was 22.5 ± 1.7 (in Soumins). Moreover, a huge cohort study with 120,700 East Asians found that, unlike Westerners, Koreans with a BMI between 22.6 and 27.5 showed the lowest risk of death [35]. Obesity is generally positively associated with oxidative stress [36]; however, our results did not show the correlation between BMI values and levels of ROS or MDA.

Our study has some limitations such as relatively small number and young age of participants. We also adopted *p* = 0.1 as the cutoff for statistical significance. Clinical studies may accept *p* < 0.1 as the threshold for statistical significance if doing so makes scientific sense but does not cause harm, as may be the case in drug tests [37]. The choice of *p* = 0.1, however, could increase the possibility of type 1 error; therefore we need to interpret our data with care.

In conclusion, our results carefully propose the differences in the serum levels of stress hormones and the oxidative stress markers across Sasang constitutional types, especially in Taeumins compared with Soumins and Soyangins. This finding would contribute to SCM-based practices by informing future research regarding the mechanisms underpinning such differences.

## Figures and Tables

**Table 1:** Characteristics of participants according to the Sasang classification.

Sex (number)	Characteristics	Taeumin	Soumin	Soyangin
Male (77)	Number (%)	18 (23.4%)	35 (45.4%)	24 (31.2%)
	Median age (yr, range)	23 (18–64)	21 (17–77)	21 (17–77)
	Average height (cm)	172.9 ± 5.2	172.6 ± 6.6	174.6 ± 5.9
	Average weight (kg)	81.7 ± 8.2	67.3 ± 7.7	73.3 ± 7.3
	Mean BMI^***^	27.3 ± 2.2	22.5 ± 1.7	24.3 ± 2.5

Female (159)	Number (%)	66 (41.5%)	43 (27.0%)	50 (31.5%)
	Median age (yr, range)	23 (18–64)	21 (17–77)	21 (17–77)
	Average height (cm)	162.6 ± 5.8	160.5 ± 4.8	159.8 ± 4.8
	Average weight (kg)	63.1 ± 12.1	51.1 ± 7.4	54.8 ± 5.4
	Average BMI^***^	24.0 ± 4.3	18.8 ± 2.6	21.4 ± 1.7

Total (236)	Number (%)	84 (35.6%)	78 (33.0%)	74 (31.4%)
	Median age (yr, range)	23 (18–64)	21 (17–77)	21 (17–77)
	Average height (cm)	164.4 ± 6.2	166.0 ± 8.3	164.6 ± 8.7
	Average weight (kg)	67.1 ± 13.7	58.4 ± 11.1	60.9 ± 10.8
	Mean BMI^***^	24.7 ± 4.2	21.0 ± 2.6	22.3 ± 2.4

^***^Comparisons of the BMIs of those with different Sasang constitutions (*p* < 0.01) were performed with ANOVAs. Those with a Taeumin constitution had significantly higher BMIs than those with a Soumin (*p* < 0.01 in male, female, and total subjects) or Soyangin (*p* < 0.01 in male, female, and total subjects) constitution, and those with a Soumin constitution had significantly lower BMIs than those with Soyangin constitution (*p* < 0.05 in male and total subjects). BMI: body mass index.
